# Whether Detection of Gene Mutations Could Identify Low- or High-Risk Papillary Thyroid Microcarcinoma? Data from 393 Cases Using the Next-Generation Sequencing

**DOI:** 10.1155/2024/2470721

**Published:** 2024-01-16

**Authors:** Lei Jin, Liang Zhou, Jian-Biao Wang, Li Tao, Xiao-Xiao Lu, Na Yan, Qian-Ming Chen, Li-Ping Cao, Lei Xie

**Affiliations:** ^1^Department of Head and Neck Surgery, Affiliated Sir Run Run Shaw Hospital, Zhejiang University School of Medicine, Hangzhou, Zhejiang, China; ^2^Key Laboratory of Digital Technology in Medical Diagnostics of Zhejiang Province, Dian Diagnostics Group Co., Ltd., Hangzhou, Zhejiang, China; ^3^Stomatology Hospital, School of Stomatology, Zhejiang University School of Medicine, Zhejiang Provincial Clinical Research Center for Oral Diseases, Hangzhou, Zhejiang, China; ^4^Department of General Surgery, Affiliated Sir Run Run Shaw Hospital, Zhejiang University School of Medicine, Hangzhou 310016, Zhejiang, China

## Abstract

**Objective:**

The objective of this study is to explore the utilization of next-generation sequencing (NGS) technology in evaluating the likelihood of identifying individuals with papillary thyroid microcarcinoma (PTMC ≤10 mm) who are at high or low risk.

**Design:**

NGS was used to analyze 393 formalin-fixed, paraffin-embedded tissues of PTC tumors, all of which were smaller than 15 mm.

**Results:**

The study found that bilateralism, multifocality, intrathyroidal spread, and extrathyroidal extension were present in 84 (21.4%), 153 (38.9%), 16 (4.1%), and 54 (13.7%) cases, respectively. Metastasis of cervical lymph nodes was identified in 226 (57.5%) cases and 96 (24.4%) cases with CLNM >5. Out of the total number of cases studied, 8 cases (2.3%) showed signs of tumor recurrence, all of which were localized and regional. Genetic alterations were detected in 342 cases (87.0%), with 336 cases revealing single mutations and 6 cases manifesting compound mutations. 332 cases (84.5%) had BRAF^V600E^ mutation, 2 cases had KRAS^Q61K^ mutation, 2 cases had NRAS^Q61R^ mutation, 8 cases had RET/PTC1 rearrangement, 3 cases had RET/PTC3 rearrangement, and 1 case had TERT promoter mutation. Additionally, six individuals harbored concurrent mutations in two genes. These mutations were of various types and combinations: BRAF^V600E^ and NRAS^Q61R^ (*n* = 2), BRAF^V600E^ and RET/PTC3 (*n* = 2), BRAF^V600E^ and RET/PTC1 (*n* = 1), and BRAF^V600E^ and TERT promoter (*n* = 1). The subsequent analysis did not uncover a significant distinction in the incidence of gene mutation or fusion between the cN0 and cN1 patient cohorts. The presence of BRAF^V600E^ mutation and CLNM incidence rates were found to be positively correlated with larger tumor size in PTMC. Our data showed that gene mutations did not appear to have much to do with high-risk papillary thyroid microcarcinoma (PTMC). However, when we looked at tumor size, we found that if the tumor was at least 5 millimeters in size, there was a higher chance of it being at high risk for PTM (*P* < 0.001, odds ratio (OR) = 2.55, 95% confidence interval (CI): 1.57–4.14). Identification of BRAF^V600E^ mutation was not demonstrated to be significantly correlated with advanced clinicopathological characteristics, although it was strongly associated with a bigger tumor diameter (OR = 4.92, 95% CI: 2.40–10.07, *P* < 0.001).

**Conclusion:**

In clinical practice, BRAFV600E mutation does not consistently serve as an effective biomarker to distinguish high-risk PTMC or predict tumor progression. The size of the tumor has a significant correlation with its aggressive characteristics. PTMC with a diameter of ≤5 mm should be distinguished and targeted as a unique subset for specialized treatment.

## 1. Introduction

Papillary thyroid carcinoma (PTC) is a pathological subtype of thyroid cancer that is the most common of all endocrine-related cancers. It is known to have low-grade, inert biological characteristics. Papillary thyroid microcarcinoma (PTMC) is defined as PTC measured ≤10 mm in its greatest diameter. In recent years, there has been a notable upward trajectory in the prevalence of PTC, with approximately half of cases diagnosed as PTMC [1–3]. However, the majority of PTMC cases are actually occult and detected due to overscreening in healthy individuals. Based on the satisfactory results from Kuma Hospital [4, 5], active surveillance (AS) has been proposed for patients with low-risk PTMC [6]. However, how to reliably and safely identify low-risk PTMC patients has noticeably attracted thyroid surgeons' attention.

Over the last two decades, substantial molecular genetic alteration studies have provided better insights into the understanding of the progression of PTC. Specific point mutations in BRAF or RAS, or the presence of RET chromosomal rearrangement, have the ability to activate the receptor tyrosine kinase (RTK)/mitogen-activated protein kinase (MAPK) pathway, a critical regulator of various cellular processes including proliferation, differentiation, adhesion, migration, and apoptosis [7–9]. PTC can progress to poorly or undifferentiated thyroid cancer by additional hits on TERT, tumor suppressors, or phosphoinositide 3-kinase (PI3K) pathway genes [8, 10–12]. In this study, we retrospectively analyzed the gene mutation status in 393 PTC patients using patients' clinical and pathological characteristics by next-generation sequencing (NGS), in order to investigate the possibility of the identification of high- or low-risk PTMC patients.

## 2. Methods

### 2.1. Patients and Samples

A total of 393 formalin-fixed, paraffin-embedded (FFPE) tumor tissues from surgically removed thyroid samples were retrospectively analyzed by the NGS between August 2018 and July 2020. All of the tumors with a maximum diameter of less than 15 mm were confirmed to be PTC by pathological examination. The postoperative follow-up periods for a total of 393 patients spanned a range from 6 to 103 months, with a median duration of 20 months. Clinical records of these individuals who underwent surgical procedures between the timeframe of February 2013 and July 2020 were meticulously reviewed utilizing a comprehensive database retrospectively compiled by the Department of Head and Neck Surgery, the Affiliated Sir Run Run Shaw Hospital. The indication of thyroidectomy was strictly in accordance with the 2009 or 2015 American Thyroid Association (ATA) guideline [13, 14]. The indications of central neck dissection (CND) and modified lateral neck dissection (LND) were previously described [15]. This research project was endorsed by the Ethical Committee of the Sir Run Run Shaw Hospital Affiliated to Zhejiang University School of Medicine.

**Figure 1:**
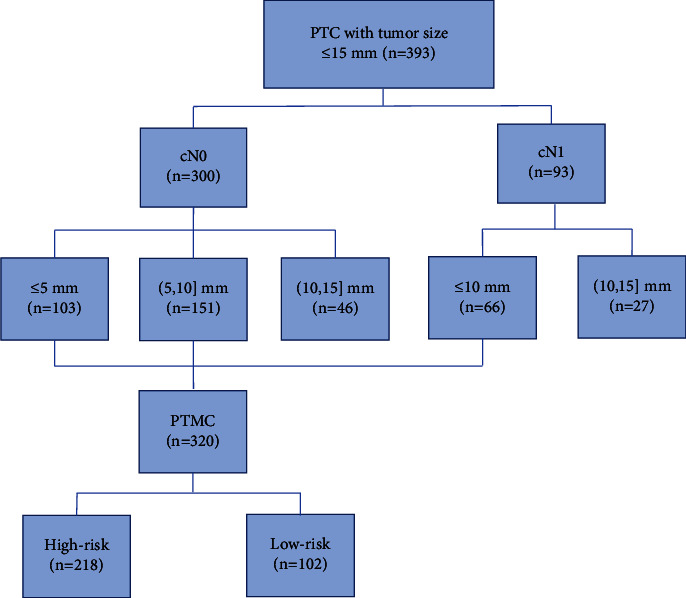
Flow diagram of included papillary thyroid carcinoma patients.

There were 300 cases with cN0, including 103 cases with a diameter of ≤5 mm, 151 cases with a diameter of 5∼10 mm, and 46 cases with a diameter of 10∼15 mm, as well as 93 cases with cN1 among 393 PTC patients. There were 320 patients with PTMC, including 218 high-risk and 102 low-risk PTMC patients (Figure 1).

### 2.2. Sample Preparation and Nucleic Acid Extraction

Following vendor manual instructions, genomic DNA and mRNA from FFPE samples were extracted using an AllPrep DNA/RNA FFPE tissue kit. This extraction kit is manufactured by Qiagen in Germany. Concentrations of DNA and cDNA were estimated using Qubit 3.0 from Thermo Fisher Scientific in the USA. In addition, DNA fragment distribution was assessed by a Qsep100 system from Bioptic, Taiwan, China.

### 2.3. NGS Library Preparation

The sequencing library should be prepared by the specific guidelines outlined in the U.S. Paragon Genomic DNA related to thyroid carcinoma was enriched by multiplex PCR, which was then connected with barcode and sequencing adapters. The amplified DNA libraries were purified using AMPure XP beads from Beckman Company of the United States. The Thermo Qubit 1 × dsDNA Assay Kit was a specific assay for quantifying next-generation sequencing libraries. The Qsep100 system developed by Biotic was used to analyze fragment size distribution. The well-known oncogenic drivers, including BRAF, RAS, TERT, and TP53, as well as gene rearrangements, such as RET and PAX-PPARG, were detected and are described in detail in Table 1.

### 2.4. Sequencing and Bioinformatics Analysis

The sequencing process was carried out on the NextSeq 500 sequencing platform from Illumina, using the PE150 sequencing strategy. Tumor samples demonstrated a mean coverage depth of approximately 1000X. The threshold for the initiation of allele mutations was set at 5%. Sequencing data were first converted to FASTQ format, and then, the human genome was mapped to the human reference sequence (hg19) using the Burrows-Wheeler aligner. Strelka was used to detect somatic insertions and deletions (InDel), while muTect was used to identify somatic single nucleotide variants (SNV). Genomic variant annotation was performed via the ANNOVAR 21 software.

### 2.5. Statistical Analysis

SPSS 20.0 software (IBM, Armonk, NY, USA) was employed for the execution of the statistical analysis. All continuous variables were reported using the mean value (standard deviation, SD) or median value (range). Categorical variables were represented as counts and corresponding percentages (%) to indicate the frequency of each category. In the event of data consistent with normal distribution and variance homogeneity, statistical analysis of differences between groups was conducted through the application of ANOVA. Mann–Whitney–Wilcoxon tests were used to evaluate differences among various groups, which were composed of data with skewed distributions. Categorical variables were assessed using either Pearson's chi-square test for large samples or Fisher's exact test for small sample sizes. To determine statistical significance, we adopted a two-sided significance threshold of *P* < 0.05 across the distribution.

## 3. Results

### 3.1. The Clinical and Pathological and Molecular Features of Tumor Size Less than 15 mm in 393 PTC Patients

This study included 393 PTC cases with tumor diameter ≥15 mm. Detailed clinicopathological features are outlined in Table 2. In the present study, 274 women and 119 men were involved, with a mean age of 43.2 ± 12.0 years. Chronic lymphocytic thyroiditis, bilateralism, multifocality, intrathyroidal spreading, and extrathyroidal extension (ETE) were found in 71 (18.1%), 84 (21.4%), 153 (38.9%), 16 (4.1%), and 54 (13.7%) patients, respectively. Cervical lymph node metastasis (CLNM) was found in 226 (57.5%) patients, including central neck compartment in 157 (40.0%) cases, lateral neck compartment in 8 (2%) cases, and both compartments in 61 (15.5%) cases; however, CLNM more than 5 (>5 CLNMs) was found in 96 (24.4%) cases. According to the 8th edition of the American Joint Committee on Cancer (AJCC) TNM staging system, 358 (91.1%) patients were classified as stage I and 35 (8.9%) patients as stage II. During the follow-up, tumor recurrence was detected in 9 (2.3%) cases, and all of them were locoregional (2 cases in contralateral lobe and 7 cases in lateral neck compartmental lymph nodes). None of them had any history of undergoing radiation therapy for head and neck cancer before surgery and distant metastasis. In addition, 3 patients were lost to follow-up.

As presented in Table 2, 332 (84.5%) cases showed BRAF^V600E^ mutation, and RAS mutation was detected in 4 (1%) cases, including KRAS^Q61K^ and NRAS^Q61R^ in 2 cases equally. All of the point mutations were missense mutations. RET/PTC rearrangement was found in 11 (2.8%) cases, including RET/PTC1 in 8 cases and RET/PTC3 in 3 cases. TERT promoter gene mutation was detected in 1 (0.3%) case. No mutations in TP53 and HRAS genes were found, and PAX8/PPARG translocation was not detected.

Besides, there were 6 cases simultaneously carrying 2 gene mutations (fusions), including BRAF^V600E^ and NRAS^Q61R^ (*n* = 2), BRAF^V600E^ and RET/PTC3 (*n* = 2), BRAF^V600E^ and RET/PTC1 (*n* = 1), and BRAF^V600E^ and TERT promoter mutation (*n* = 1). No one had three or more gene mutations (fusions). However, it is noteworthy that 5 patients had older ages (>60 years old), and all of their PTCs had low-risk manifestations. The clinicopathological data of these 6 patients are listed in Table 3.

### 3.2. Comparison of Gene Mutation Status and Clinical Pathological Characteristics between cN0 and cN1 Patients with Tumor Diameter Less than 15 mm

Among 393 PTC patients, 93 (23.7%) patients showed clinical CLNM (cN1) before surgery. The 393 PTC patients whose tumor size was less than 15 mm were dispensed into two groups: cN0 group (*n* = 300) and cN1 (*n* = 93) group. Group cN1 was regarded as simulated clinical progression of observable PTC with cN0 (≤15 mm in size). Table 2 compares the clinicopathological features and genetic alterations between the cN0 and cN1 groups.

Compared with cN0 PTC, cN1 PTC showed more aggressive features, such as maximum tumor size, bilateralism, multifocality, intrathyroidal spreading, ETE, and LNM, except for TNM staging and tumor recurrence. Nevertheless, genetic mutations or fusions observed between cN0 and cN1 groups were not significantly different for BRAF^V600E^, NRAS^Q61R^, KRAS^Q61K^, TERT promoter, and RET/PTC.

### 3.3. Correlation Analysis of Tumor Growth with Clinicopathological Features and Gene Mutation Status in cN0 PTC Patients with Tumor Size Less than 15 mm

In order to gain insight into the genetic mutation status during the progression of PTC, a total of 300 cN0 PTC cases were categorized into three parts based on primary tumor diameter: the first group (group A) consisted of 103 patients with tumor diameters of 5 mm or less, while the second group (group B) had a total of 151 patients with tumor diameters ranging from 5 mm to 10 mm. The third group (group C) contained 46 patients with tumor diameters ranging from 10 mm to 15 mm. Clinical progression of observable PTMCs was simulated by evaluating group C. The data are shown in Table 4.

As to the clinicopathological features, only CLNM was significantly different among the three groups (group A vs. group B, *P* < 0.001, odds ratio (OR) = 0.32; group B vs. group C, *P*=0.047, OR = 0.49), including >5 CLNMs (group A vs. group B, *P* < 0.001, OR = 0.06; group B vs. group C, *P*=0.301, OR = 0.65). Regarding genetic mutation, there was a significant difference only in BRAF^V600E^ mutation among the three groups (group A vs. group B, *P* < 0.001, OR = 0.23; group B vs. group C, *P*=0.369, OR = 0.45). Therefore, the incidence rates of CLNM and BRAF^V600E^ mutation could increase during the PTMC growth.

### 3.4. Perform Univariate and Multivariate Logistic Regression Analyses on PTMC High-Risk Factors

All PTMC patients were divided into two groups: low-risk group (*n* = 102) and high-risk group (*n* = 218). It was found that BRAF^V600E^, NRAS^Q61R^, KRAS^Q61K^, RET/PTC1, and RET/PTC3 showed no correlation with high-risk PTMC (Table 5). The multivariate logistic regression analysis has been further confirmed that gene mutations were not risk factors for high-risk PTMC in the present study; however, a significant correlation was identified between bigger tumor diameter and high-risk PTMC (*P* < 0.001, OR = 2.55, 95% confidence interval (CI): 1.57–4.14).

As per the ATA guidelines published in 2015, patients with more than 5 CLNMs are classified as the non-low-risk category. Therefore, the multivariate logistic regression analysis was carried out to appraise the integrated effect of histopathological and molecular characteristics on >5 CLNMs. It was revealed that gene mutations, including single and multiple mutations, were not significantly associated with >5 CLNMs, whereas male gender (*P*=0.001, OR = 3.1, 95% CI: 1.60–5.86), older age at the time of diagnosis (40–60 years old, *P* < 0.001, OR = 3.73, 95% CI: 1.91–7.28; >60 years old, *P*=0.006, OR = 17.89, 95% CI: 2.27–140.10), larger tumor size (*P* < 0.001, OR = 5.25, 95% CI: 2.29–12.04), intrathyroidal spreading (*P*=0.007, OR = 10.62, 95% CI: 1.88–59.80), and bilateralism (*P*=0.016, OR = 2.47, 95% CI: 1.18–5.18) were significantly associated with >5 CLNMs (Table 6).

### 3.5. The Association of the Presence of BRAF^V600E^ Mutation with Clinicopathological Features of PTMC Patients

As the prevalence of BRAF^V600E^ mutation was significantly higher than the other mutations in PTC patients, it was further attempted to analyze the association between BRAF^V600E^ mutation and clinicopathological features of PTMC patients. It was found that there was not a big connection between the presence of BRAF^V600E^ mutation and the more serious stages of the disease, such as multifocality, intrathyroidal spreading, ETE, LNM, and tumor recurrence, while it was significantly associated with the bigger tumor diameter (OR = 4.92, 95% CI: 2.40–10.07, *P* < 0.001) (Table 7).

## 4. Discussion

In a 30-year longitudinal study of 140 cases, Woolner et al. established the concept of occult papillary carcinoma in 1960, referring to PTCs less than or equal to 15 mm in size [16]. Back in 1989, the World Health Organization created a new terminology to replace occult papillary carcinoma. They named it papillary microcarcinoma and decided it should be defined as papillary thyroid cancer that is less than or equal to 10 mm in diameter [17]. PTMC has been recognized as an essential variation of PTC due to exhibiting minimal malignant potential and rarely undergoing distant metastasis. These characteristics are typically found in autopsy studies at a frequency ranging from 6% to 35%. However, modern diagnostic methods have increased the detection rate of this type of cancer in the living. The best diameter of low-risk PTMCs for observation is usually between 10 mm and 15 mm. However, the Chinese Association of Thyroid Oncology (CATO) recommends that the dimensions of low-risk papillary thyroid microcarcinomas identified should not exceed 5 mm [18]. The perspective of CATO is derived from a comprehensive examination of two discriminating markers for AS in a sample of 1,001 low-risk patients diagnosed with PTMC. Compared to the findings in the Kuma low-risk PTMC group (lesions ≤10 mm), Qian et al. observed a significantly lower incidence of multifocal lesions, extrathyroidal extensions (ETEs), central LN metastasis, and progression rates in the CATO low-risk PTMC group (lesions ≤5 mm). Moreover, there was an observed improvement in the duration of disease-free survival. So we selected PTC patients with primary tumors that were less than or equal to 15 mm. We divided these patients into three groups based on their tumor size. The tumors that measured from 10 mm to 15 mm were presumed to represent a clinical progression of the visually noted PTMC.

From the study of the PTC in The Cancer Genome Atlas (TCGA) cohort that excluded poorly differentiated and anaplastic thyroid cancers, alteration of the MAPK pathway was detected in 83% of all the tested PTC samples, with the mutually exclusive activating mutations in BRAF (62%) and RAS (13%), as well as RET/PTC rearrangement (6%), and less frequently NTRK3 fusion (1.5%), NTRK1 fusion (1.3%), and ALK fusion (0.8%) [19]. Regarding PTMC, Rodrigues et al. reviewed 49 studies published from 1998 to 2015 and summarized the overall prevalence of BRAF^V600E^, RAS, TERT promoter mutation, and RET/PTC rearrangement in 57.4% (5741/10004), 3.8% (4/106), 4.6% (19/409), and 44% (70/159) of cases, respectively [20]. In the present study, the prevalence of BRAF^V600E^, RAS, TERT promoter mutation, and RET/PTC rearrangement in PTC samples with tumor size ≤15 mm was 84.5% (332/393), 1.0% (4/393), 0.3% (1/393), and 2.8% (11/393), respectively; however, in PTMC samples, no TERT promoter mutation was detected, and the prevalence of BRAF^V600E^, RAS mutation, and RET/PTC rearrangement was 82.8% (265/320), 0.9% (3/320), and 2.5% (8/320), respectively. The difference in the spectrum of genetic alterations mentioned above was supposed to be associated with race, patient selection, the gene sequencing technique, the source of specimens, and the histologic subtype of PTC.

BRAF, RAS, and RET/PTC mutations are commonly recognized as mutually exclusive in PTC. This suggests that more than one mutation in these genes would likely not provide any additional biological benefits beyond those already conferred by a single mutation [21–23]. Zou et al. reported concomitant BRAF^V600E^ and KRAS^Q61R^ mutations in 2 classical PTCs, concomitant BRAF^V600E^ mutation and RET/PTC1 rearrangement in 6 classical PTCs, and 1 tall-cell variant of PTC as well as concomitant NRAS^Q61R^ mutation and RET/PTC1 rearrangement in 2 follicular variants of PTCs, and they demonstrated that concomitant gene mutation might be the cause of intratumor heterogeneity. Furthermore, they found that the concomitant mutations were associated with the advanced stage of the disease [24]. In the present study, there were 6 PTC cases simultaneously carrying 2 gene mutations, including BRAF^V600E^ and NRAS^Q61R^ (*n* = 2), BRAF^V600E^ and RET/PTC3 (*n* = 2), BRAF^V600E^ and RET/PTC1 (*n* = 1), and BRAF^V600E^ and TERT promoter mutation (*n* = 1). However, such cases showed no noticeable aggressive tumor behaviors. Among these 6 patients, it was found that 5 patients had older age (> 60 years old); thus, we hypothesized that with the increase of age, the effects of carcinogenic factors on the body were enhanced, which might lead to multiple mutations or fusion of somatic genes.

It is recommended at Kuma Hospital that low-risk patients with PTMC undergo active surveillance (AS) as the primary treatment [6]. In the event that a tumor exhibits an increase in size of 3 mm or more, a tumor measuring 12 mm in size, or the presence of lymph node metastases during the follow-up period, prompt consideration should be given to the implementation of “rescue surgery.” What role does gene mutation play in the course of disease progression? Whether gene mutation could predict the progression of PTMC? In light of these observations, we employed our dataset to model the progression of clinical symptoms and designed two distinct segments. In the first part, it was attempted to simulate LNM using two groups of cN0 and cN1, according to whether LNM was confirmed preoperatively. Expectedly, more aggressive features of PTMC were found in the cN1 group, while gene mutation showed no significant difference between the two groups. In the second part, cN0 patients were divided into three groups: ≤5 mm, 5∼10 mm, and 10∼15 mm according to the tumor size, in order to simulate tumor growth. It was found that the incidence of BRAF^V600E^ mutation significantly increased during the growth of PTMC, suggesting that BRAF^V600E^ mutation might promote tumor growth. The mechanism may include three aspects: (1) the BRAF^V600E^ mutation leads to sustained activation of the RAS-RAF-MEK-ERK/MAPK pathway, enhancing cell mitotic capacity, ultimately leading to abnormal cell proliferation, and inducing tumor occurrence [10]. (2) The BRAF^V600E^ mutation promotes methylation of the thyrotropin receptor (TSHR) gene promoter, leading to TSHR silencing or a significant decrease in expression, increasing the feedback of thyrotropin (TSH), and promoting tumor cell growth [25]. (3) The BRAF^V600E^ mutation upregulates TERT expression in a non-TERT promoter-dependent manner through the oncogene MYC, which also promotes the occurrence of PTC [26]. However, in clinical practice, BRAFV600E mutation could be used as a biomarker for identifying thyroid nodule malignancy, rather than predicting tumor growth.

Multiple studies have documented a favorable prognosis among patients with PTMC [27–30], with results indicating a clinical course that is generally favorable. This favorable prognosis has been corroborated by prospective clinical studies, in which patients with PTMC who chose not to undergo surgical intervention received AS primarily [31, 32]. Zaid et al. reported a series of 30,180 adult patients with PTMC. Notably, 5,628 patients (18.7%) showed advanced features, including central lymph node metastasis (8.0%), lateral lymph node metastasis (4.4%), microscopic extrathyroidal extension (6.7%), macroscopic extrathyroidal extension (0.3%), lymphovascular invasion (4.4%), and distant metastasis (0.4%). They described PTMC as a wolf in sheep's clothing and suggested that lobectomy seemed to be a more appropriate treatment for low-risk PTMC patients rather than AS [33]. Whether gene detection can identify low- or high-risk PTMC patients preoperatively? PTMC was dispensed into two groups in our study: the high-risk group, in which PTMC had some advanced features, including ETE, LNM, multifocality, and intrathyroidal spreading, and the low-risk group (without any aggressive features). As a result, it was revealed that the larger the tumor size, the more aggressive the characteristics, and only tumor size could affect the advanced features of PTMC, rather than gene mutation, especially BRAF^V600E^.

Although the primary objective of this study was to explore the correlation between gene mutations and the aggressiveness of the PTMC, it was clearly found that the tumor size, rather than gene mutation, was significantly associated with the progression of high-risk PTMC. For instance, when the tumor grew up from 5 to 10 mm, the incidence of LNM was doubled, and the nonmutation rate of BRAF^V600E^ was tripled. The findings of the present study were also similar to the previously reported results. After analysis of 977 PTMC cases, Zheng et al. found that tumor size (>5 mm) was an independent risk factor associated with LNM [34]. In a study looking at two screening criteria for AS, Qian et al. found that low-risk patients with less than 5 mm of PTMC had a lower chance of having multifocal lesions, ETE, central LNM, or disease progression. This means they were also less likely to relapse and had longer disease-free survival times [18]. Therefore, a tumor with a size of less than 5 mm should be regarded as a special subgroup of PTMC that showed to be less aggressive than the other PTMCs and might be treated specially with local ablation, such as radiofrequency, in order to block the tumor progression during the follow-up of PTMC.

In conclusion, our findings indicate that BRAFV600E mutation may not be a reliable biomarker to clinically distinguish high-risk PTMC patients or predict tumor progression. Tumor size was found to be highly correlated with the disease's aggressiveness. PTMC tumors measuring ≤5 mm should be identified as a distinct subgroup and treated with specific treatment strategies.

### 4.1. Limitation

The present study had several limitations. First, the 393 patients in this cohort were analyzed inconsecutive from 2013 to 2020 because we were unable to obtain consent from all patients for NGS of their samples. The other reason was that not all patient samples were of suitable quality for NGS. Therefore, a selective bias might exist in the study. Second, the relatively short follow-up time may have an impact on the long-term recurrence rate. In our study, tumor recurrence was detected in 9 (2.3%) cases, and it may be better than other studies, but we believe the data are true. From January 2013 to December 2015, our medical center treated a total of 562 patients with papillary thyroid cancer (PTC) with initial surgical therapy during our prior research endeavor, which consisted of either unilateral lobectomy or total thyroidectomy, and central neck dissection. Some patients also underwent lateral neck dissection. Five recurrence cases were identified between a minimum follow-up period of 40 months and a maximum follow-up period of 75 months, resulting in a median follow-up period of 57 months [35]. We speculate that the recurrence rate is lower than others because all the patients underwent prophylactic or therapeutic cervical lymph node dissection, and the surgical team is very experienced and well-practiced. Third, samples for the NGS were from formalin-fixed, paraffin-embedded tumor tissues and histologic subtypes of PTC were not further classified; therefore, the map of gene mutation might be slightly different from other relative studies. Lastly, this NGS panel was small, but it included 9 hot spot gene mutations and rearrangements related to thyroid cancer and is a cost-effective auxiliary diagnostic test for thyroid nodules. The other PTC-associated drivers, such as EIF1AX, AKT1, and PIK3CA, will be considered and measured in future research.

## Figures and Tables

**Table 1:** Genetic testing site in the study.

Gene	Testing site
BRAF	Val600

KRAS	Codon12/13/59/61/146

NRAS	Codon12/13/59/61/117/146

HRAS	Codon 12/13/61

TERT	C228T/C250T

TP53	Arg273

PAX8/PPARG	PAX8{NM_003466.2}: r.1_943_PPARG{NM_015869.2}: r.216_1854
	PAX8{NM_003466.2}: r.1_1064_PPARG{NM_015869.2}: r.216_1854
	PAX8{NM_003466.2}: r.1_1253_PPARG{NM_015869.2}: r.216_1854
	PAX8{NM_003466.2}: r.1_1355_PPARG{NM_015869.2}: r.216_1854

CCDC6/RET	CCDC6{ENST00000263102}: R.1_535_RET{ENST00000355710}: r.2369_5659

NCOA4/RET	NCOA4{ENST00000452682}: R.1_1014_RET{ENST00000355710}: r.2369_5659

**Table 2:** Clinicopathological characteristics and genetic alterations of 393 PTCs subdivided into cN0 and cN1 groups.

	Total	cN0	cN1	*P*
	*n* = 393	*n* = 300	*n* = 93	
Gender				
M	119 (30.3%)	83 (27.7%)	36 (38.7%)	**0.043**
F	274 (69.7%)	217 (72.3%)	57 (61.3%)	
Age at diagnosis (years)	43.2 ± 12.0	43.8 ± 12.0	41 ± 11.9	**0.048**
Maximum tumor size (mm)				
Mean ± SD	7.6 ± 3.3	7.3 ± 3.2	8.8 ± 3.4	<**0.001**
Chronic lymphocytic thyroiditis				
Yes	71 (18.1%)	47 (15.7%)	24 (25.8%)	**0.026**
No	322 (81.9%)	253 (84.3%)	69 (74.2%)	
Bilateralism				
Yes	84 (21.4%)	50 (16.7%)	34 (36.6%)	<**0.001**
No	309 (78.6%)	250 (83.3%)	59 (63.4%)	
Multifocality				
Yes	153 (38.9%)	105 (35.0%)	48 (51.6%)	**0.004**
No	240 (61.1%)	195 (65.0%)	45 (48.4%)	
Intrathyroid spread				
Yes	16 (4.1%)	6 (2.0%)	10 (10.8%)	**0.001**
No	377 (95.9%)	294 (98.0%)	83 (89.2%)	
Extrathyroidal extension				
Yes	54 (13.7)	35 (11.7%)	19 (20.4%)	**0.032**
No	339 (86.3%)	265 (88.3%)	74 (79.6%)	
Lymph node metastases				
No	167 (42.5%)	161 (53.7%)	6 (6.4%)	<**0.001**
Central compartment	157 (40.0%)	139 (46.3%)	18 (19.4%)	<**0.001**
Laterocervical compartment	8 (2%)	0 (0%)	8 (8.6%)	<**0.001**
Central +	61 (15.5%)	0 (0%)	61 (65.6%)	<**0.001**
Latercervical compartment				
>5	96 (24.4%)	34 (11.3%)	62 (66.7%)	<**0.001**
≤5	297 (75.6%)	266 (88.7%)	31 (33.3%)	
Staging (VIII ed. TNM)				
I	358 (91.1%)	277 (92.3%)	81 (87.1%)	0.121
II	35 (8.9%)	23 (7.7%)	12 (12.9%)	
Tumor recurrence				
Yes	9 (2.3%)	5 (1.7%)	4 (4.3%)	0.225
No	381 (97.0%)	293 (97.7%)	88 (94.6%)	0.165
n.a.	3 (0.7%)	2 (0.6%)	1 (1.0%)	0.556
BRAF^V600E^ mutation				
Yes	332 (84.5%)	252 (84.0%)	80 (86%)	0.638
No	61 (15.5%)	48 (16.0%)	13 (14%)	
KRAS mutation				
wt.	391 (99.5%)	299 (99.7%)	92 (98.9%)	0.418
Q61K	2 (0.5%)	1 (0.3%)	1 (1.1%)	
NRAS mutation				
wt.	391 (99.5%)	298 (99.3%)	93 (100%)	1.000
Q61R	2 (0.5%)	2 (0.7%)	0 (0%)	
RET/PTC				
wt.	382 (97.2%)	294 (98.0%)	88 (94.6%)	0.140
RET/PTC1	8 (2.0%)	5 (1.7%)	3 (3.2%)	0.400
RET/PTC3	3 (0.8%)	1 (0.3%)	2 (2.2%)	0.141

wt: wild type, na: not available, and PTC: papillary thyroid carcinoma. Clinically apparent metastatic disease to nodes (cN1) was defined as LN metastasis in the central or lateral neck suspected by ultrasonography or computed tomography and proved by ultrasound-guided fine needle aspiration (FNA) and the washout of thyroglobulin preoperatively. Data are expressed as mean ± SD or absolute number (%). Comparison between cN0 and cN1 groups was performed by ANOVA or chi-square test when appropriate. Significant differences are shown in bold characters (*P* < 0.05).

**Table 3:** The clinicopathological characteristics of PTC patients simultaneously carried two gene mutations (fusions).

No.	Genetic alteration	Gender	Age (years)	Size (mm)	CLT	Bilateralism	Multifocality	ITS	ETE	Lymph node metastases			Tumor recurrence
										Total	Central compartment	Laterocervical compartment	
1	BRAF^V600E^ NRASQ^61R^	M	48	13	No	No	Yes	No	No	0/28	0/8	0/20	No
2	BRAF^V600E^ NRASQ^61R^	F	64	6	No	No	No	No	No	0/8	0/8	—	No
3	BRAF^V600E^ RET/PTC1	M	70	10	No	No	No	No	No	0/8	0/8	—	No
4	BRAF^V600E^ RET/PTC3	M	61	5	No	No	No	No	No	2/14	2/14	—	No
5	BRAF^V600E^ RET/PTC3	F	63	5	No	No	No	Yes	No	4/31	2/8	2/23	No
6	BRAF^V600E^ TERT promoter	M	84	13	No	No	No	No	No	0/7	0/7	—	No

CLT: chronic lymphocytic thyroiditis; ITS: intrathyroid spread; ETE: extrathyroidal extension.

**Table 4:** Patient characteristics and genetic alterations of cN0 PTCs (*n* = 300).

	Total	≤5 mm	(5, 10]mm	(10, 15]mm	*P*
	*n* = 300	*n* = 103	*n* = 151	*n* = 46	
Gender					
M	83 (27.7%)	25 (24.3%)	39 (25.8%)	19 (41.3%)	0.077
F	217 (72.3%)	78 (75.7%)	112 (74.2%)	27 (58.7%)	
Age at diagnosis (years)	43.9 ± 12.0	44.8 ± 10.9	43.8 ± 12.6	41.9 ± 12.2	0.406
Maximum tumor size (mm)					
Mean ± SD	7.3 ± 3.2	3.9 ± 1.1	7.9 ± 1.5	12.7 ± 1.3	<**0.001**
Chronic lymphocytic thyroiditis					
Yes	47 (15.7%)	15 (14.6%)	24 (15.9%)	8 (17.4%)	0.903
No	253 (84.3%)	88 (85.4%)	127 (84.1%)	38 (82.6%)	
Bilateralism					
Yes	50 (16.7%)	13 (12.6%)	23 (15.2%)	14 (30.4%)	0.061
No	250 (83.3%)	90 (87.4%)	128 (84.8%)	32 (69.6%)	
Multifocality					
Yes	105 (35.0%)	30 (29.1%)	53 (35.1%)	22 (47.8%)	0.087
No	195 (65.0%)	73 (70.9%)	98 (64.9%)	24 (52.2%)	
Intrathyroid spread					
Yes	6 (2.0%)	1 (1.0%)	2 (1.3%)	3 (6.5%)	0.088
No	294 (98.0%)	102 (99.0%)	149 (98.7%)	43 (93.5%)	
Extrathyroidal extension					
Yes	35 (11.7%)	8 (7.8%)	21 (13.9%)	6 (13.0%)	0.310
No	265 (88.3%)	95 (92.2%)	130 (86.1%)	40 (87.0%)	
Lymph node metastases					
No	161 (53.7%)	76 (73.8%)	71 (47.0%)	14 (30.4%)	<**0.001**
Yes (central compartment)	139 (46.3%)	27 (26.2%)	80 (53.0%)	32 (69.6%)	
>5	34 (11.3%)	1 (1.0%)	23 (15.2%)	10 (21.7%)	<**0.001**
≤5	266 (88.7%)	102 (99.0%)	128 (84.8%)	36 (78.3%)	
Staging (VIII ed. TNM)					
I	277 (92.3%)	97 (94.2%)	138 (91.4%)	42 (91.3%)	0.699
II	23 (7.7%)	6 (5.8%)	13 (8.6%)	4 (8.7%)	
Tumor recurrence					
Yes	5 (1.7%)	1 (1.0%)	2 (1.3%)	2 (4.3%)	0.299
No	293 (97.7%)	102 (99.0%)	148 (98.0%)	43 (93.5%)	0.135
n.a.	2 (0.6%)	0 (0%)	1 (0.7%)	1 (2.2%)	0.401
BRAF^V600E^ mutation					
Yes	252 (84.0%)	71 (68.9%)	137 (90.7%)	44 (95.7%)	<**0.001**
No	48 (16.0%)	32 (31.1%)	14 (9.3%)	2 (4.3%)	
KRAS mutation					
wt.	299 (99.7%)	102 (99.0%)	151 (100%)	46 (100%)	0.497
Q61K	1 (0.3%)	1 (1.0%)	0 (0%)	0 (0%)	
NRAS mutation					
wt.	298 (99.3%)	103 (100%)	150 (99.3%)	45 (97.8%)	0.401
Q61R	2 (0.7%)	0 (0%)	1 (0.7%)	1 (2.2%)	
RET/PTC					
wt.	294 (98.0%)	101 (98.0%)	148 (98.0%)	45 (97.8%)	1.000
RET/PTC1	5 (1.7%)	1 (1.0%)	3 (2.0%)	1 (2.2%)	0.709
RET/PTC3	1 (0.3%)	1 (1.0%)	0 (0%)	0 (0%)	0.497

wt: wild type, na: not available, and PTC: papillary thyroid carcinoma. Data are expressed as mean ± SD or absolute number (%). *P* value means statistical analysis among the three groups by ANOVA or chi-square test. Data are expressed as mean ± SD or absolute number (%). Significant differences are shown in bold characters (*P* < 0.05).

**Table 5:** Patient characteristics of 320 PTMC divided into low- and high-risk groups.

	Total	Low-risk	High-risk	*P*
	*n* = 320	*n* = 102	*n* = 218	
Gender				
M	86 (26.9%)	24 (23.5%)	62 (28.4%)	0.417
F	234 (73.1%)	78 (76.5%)	156 (71.6%)	
Age at diagnosis (years)	43.4 ± 11.9	44.2 ± 11.7	43 ± 11.7	0.389
Maximum tumor size (mm)				
Mean ± SD	6.4 ± 2.3	5.6 ± 2.2	6.8 ± 2.3	<**0.001**
≤5	118 (35%)	53 (52.0%)	65 (29.8%)	<**0.001**
>5	202 (65%)	49 (48.0%)	153 (70.2%)	
Chronic lymphocytic thyroiditis				
Yes	56 (17.5%)	17 (16.7%)	39 (17.9%)	0.875
No	264 (82.5%)	85 (83.3%)	179 (82.1%)	
Staging (VIII ed. TNM)				
I	295 (92.2%)	102 (100%)	193 (88.5%)	<**0.001**
II	25 (7.8%)	0	25 (11.5%)	
Tumor recurrence				
Yes	7 (2.2%)	1 (1.0%)	6 (2.8%)	0.437
No	311 (97.2%)	101 (99.0%)	210 (96.3%)	0.281
n.a.	2 (0.6%)	0	2 (0.9%)	1.000
BRAF				
Wt.	55 (17.2%)	15 (14.7%)	40 (18.3%)	0.525
V600E	265 (82.8%)	87 (85.3%)	178 (81.7%)	
KRAS				
wt.	318 (99.4%)	101 (99.0%)	217 (99.5%)	0.537
Q61K	2 (0.6%)	1 (1.0%)	1 (0.5%)	
NRAS				
wt.	319 (99.7%)	101 (99.0%)	218 (100%)	0.319
Q61R	1 (0.3%)	1 (1.0%)	0	
RET/PTC				
wt.	312 (97.5%)	100 (98%)	212 (97.2%)	1.000
RET/PTC1	5 (1.6%)	2 (2%)	3 (1.4%)	0.655
RET/PTC3	3 (0.9%)	0	3 (1.4%)	0.554
BRAF + NRAS				
Yes	1 (0.3%)	1 (1.0%)	0	0.319
No	319 (99.7%)	101 (99.0%)	218 (100%)	
BRAF + RET/PTC				
Yes	3 (0.9%)	1 (1.0%)	2 (0.9%)	1.000
No	317 (99.1%)	101 (99%)	216 (99.1%)	

wt: wild type, na: not available, and PTMC: papillary thyroid microcarcinoma. The high-risk PTMC was defined as having one or more advanced characteristics including multifocality, extrathyroidal extension (ETE), intrathyroid spread, and LN metastasis, whereas the low-risk PTMC has none of these features. Data are expressed as mean ± SD or absolute number (%). Comparison between low-risk and high-risk groups was performed by ANOVA or chi-square test when appropriate. Significant differences are shown in bold characters (*P* < 0.05).

**Table 6:** Multivariate logistic regression analyses of more than 5 cervical lymph node metastases in 320 PTMC.

Independent variable	OR	CI 95%	*P*
Gender			
F	1	—	Ref.
M	3.1	1.60–5.86	**0.001**
Age at diagnosis (years)			
≤40	1	—	Ref.
(40, 60]	3.73	1.91–7.28	**<0.001**
>60	17.89	2.27–140.10	**0.006**
Maximum tumor size (mm)			
≤5	1	—	Ref.
>5	5.25	2.29–12.04	**<0.001**
Intrathyroid spread			
No	1	—	Ref.
Yes	10.62	1.88-59.80	**0.007**
Bilateralism			
No	1	—	Ref.
Yes	2.47	1.18–5.18	**0.016**

CI: confidence interval, OR: odds ratio, and PTMC: papillary thyroid microcarcinoma. *p* < 0.05 is significant.

**Table 7:** Multinomial logistic regression analysis to evaluate the association between the presence of BRAF^V600E^ and patient characteristics of PTMC.

Independent variable	OR	CI 95%	*P*
Gender			
F	1	—	Ref.
M	1.49	0.66–3.37	0.324
Age at diagnosis (years)			
≤40	0.46	0.07–3.00	0.455
(40, 60]	0.24	0.04–1.46	0.089
>60	1	—	Ref.
Maximum tumor size (mm)			
≤5	1	—	Ref.
>5	4.92	2.40-10.07	<**0.001**
Chronic lymphocytic thyroiditis			
No	1	—	Ref.
Yes	0.7	0.31–1.60	0.401
Bilateralism			
No	1	—	Ref.
Yes	0.6	0.19–1.90	0.686
Multifocality			
No	1	—	Ref.
Yes	5.12	1.48-17.70	0.996
Intrathyroid spread			
No	1	—	Ref.
Yes	1.02	0.10-10.25	0.67
Extrathyroidal extension			
No	1	—	Ref.
Yes	2.39	0.73–7.78	0.749
Lymph node metastases			
No	1	—	Ref.
Central compartment	2.53	0.78–8.21	0.481
Laterocervical compartment	8	0.65–98.99	0.627
Central +	2.9	0.54-15.57	0.985
Laterocervical compartment			
≤5	1	—	Ref.
>5	0.85	0.23–3.09	0.625
Staging (VIII ed. TNM)			
I	1	—	Ref.
II	3.68	0.54-25.15	0.256
Tumor recurrence			
Yes	0.66	0.51–8.60	0.877
No	1	—	Ref.
n.a.	0.03	0.00–0.59	0.048

na: not available and PTMC: papillary thyroid microcarcinoma. Significant differences are shown in bold characters (*P* < 0.05).

## Data Availability

The raw data reported in this study can be accessed and downloaded from the CNGB Nucleotide Sequence Archive (CNSA) (https://db.cngb.org/cnsa/) with accession no. CNP0004004.
